# Molecular xenomonitoring as a post-MDA surveillance tool for global programme to eliminate lymphatic filariasis: Field validation in an evaluation unit in India

**DOI:** 10.1371/journal.pntd.0007862

**Published:** 2020-01-24

**Authors:** Swaminathan Subramanian, Purushothaman Jambulingam, Kaliannagounder Krishnamoorthy, Neelavathi Sivagnaname, Candasamy Sadanandane, Venkatesan Vasuki, Chokkalingam Palaniswamy, Balakrishnan Vijayakumar, Adinarayanan Srividya, Hari Kishan K. Raju

**Affiliations:** 1 ICMR-Vector Control Research Centre, Indira Nagar, Puducherry, India; 2 Office of the Deputy Director of Health Services, Department of Public Health, Cuddalore, Tamil Nadu, India; Faculty of Science, Ain Shams University (ASU), EGYPT

## Abstract

**Background:**

Lymphatic filariasis (LF) is targeted for elimination by the year 2020. As of 2017, 67 of the 72 endemic countries have implemented annual Mass Drug Administration (MDA) for interrupting LF transmission. Transmission Assessment Survey (TAS) is the recommended protocol to evaluate the impact of MDA and to decide when to stop MDA in an Evaluation Unit (EU, population ≤2 million). As the human infection levels go down with repeated MDA rounds, it becomes a challenge to select the appropriate survey methods to assess transmission interruption. This study validates a standard protocol for molecular xenomonitoring of infection in vectors (MX) at an EU as a complementary tool for TAS to stop MDA and its utility for post-MDA or post-validation surveillance.

**Methodology:**

The study was conducted in Cuddalore district, Tamil Nadu, India, which was found eligible for TAS after 15 annual rounds of MDA (4 with DEC alone and 11 with DEC plus albendazole). The district was divided into two EUs as per the TAS protocol and one EU was randomly selected for the study. A two-stage cluster design vector sampling, developed and validated at a sub-district level, was implemented in 30 randomly selected clusters in the EU. Female *Culex quinquefasciatu*s were collected placing gravid traps overnight (1800–0600 hrs) inside the premises of systematically selected households. Pools of 20–25 blood-fed, semi-gravid and gravid *Cx*. *quinquefasciatus* were subjected to real-time quantitative PCR (polymerase chain reaction) assay for detecting *Wuchereria bancrofti* DNA. Pool infection rate (% of pools positive for *W*. *bancrofti* DNA), and the estimated prevalence of *W*. *bancrofti* DNA in mosquitoes and its 95% confidence interval were calculated. Additionally, in these 30 clusters, microfilaria (Mf) survey among individuals >5 years old was carried out. School-based TAS was conducted using Immunochromatographic Card Test (ICT) in the EU. Prepared itemized cost-menu for different cost components of MX survey and TAS were estimated and compared.

**Results:**

MX survey showed that only 11 (3.1%) of the 358 pools (8850 *Cx*.*quinquefasciatus* females), collected from 30 clusters, were found positive for *W*. *bancrofti* DNA. The estimated vector infection rate was 0.13% (95% CI: 0.07–0.22%), below the provisional threshold (0.25%) for transmission interruption. Of 1578 children tested in the TAS, only four (0.25%) were positive for filarial antigenemia, and it is well below the critical cut-off (18 positives) for stopping MDA. Among 9804 persons tested in the 30 clusters, only four were found positive for Mf (0.04%; 95% CI: 0.01–0.1%). The Mf-prevalence was <1% threshold for transmission interruption in humans. The estimated costs for TAS and MX per EU were $14,104 USD and $14,259 USD respectively.

**Conclusions:**

The result of MX protocol was in good agreement with that of TAS, providing evidence to recommend MX as a complementary tool to TAS to decide on stopping MDA. MX can also be a potential surveillance tool for post-MDA and post-validation phases as it could detect sites with residual infection and risk of resurgence of transmission. MX is economically feasible as its cost is slightly higher than that of TAS.

## Author summary

Lymphatic filariasis (LF), commonly known as “elephantiasis” is caused by filarial parasites and transmitted among humans by mosquitoes. This parasitic infection results in chronic diseases such as swelling of limbs and hydrocele. Global programme to eliminate lymphatic filariasis (GPELF), launched by the World Health Organization (WHO) in 2000 endorsed the mass treatment of all the people above 2 years of age in the endemic areas with a single dose of anti-filarial drugs administered annually for a minimum period of 5 years. WHO also recommended transmission assessment survey (TAS) protocol to assess the impact of mass treatment and to decide on stopping mass treatment. The protocol aims at screening young children who were born after the mass treatment for filarial infection. If the number of infected children is smaller than the pre-defined number, mass treatment can be stopped. The same protocol is followed for periodical assessment to verify whether there are any new infections. Alternatively, vector infection levels by molecular xenomonitoring (MX, detection of parasite DNA in the mosquitoes) can be used to verify whether there are any infected mosquitoes. This tool has been applied in many studies and there is a provisionally established mosquito infection threshold level (0.25%) below which transmission is interrupted. This can be an alternative tool for TAS. We validated this method at district level by collecting filariasis transmitting mosquitoes from 30 villages/wards and compared the results with those of TAS. There was good agreement between the decisions based on TAS and MX in our study. Though in the EU both vector and human infection levels were below their respective threshold levels, the mosquito infection in individual sites was above the threshold, indicating residual hotspots and risk of resurgence. In addition, we estimated the cost of conducting MX and TAS for their economic feasibility and found that the cost of MX is only marginally higher than that of school-based TAS. Thus, our study results provide recommendations to use MX as a tool complementary to TAS (i) for taking a decision on stopping MDA, (ii) for monitoring post-MDA and post-validation surveillance programme, and (iii) for remapping areas to initiate MDA.

## Introduction

The global programme to eliminate lymphatic filariasis (GPELF) launched in 2000 with the goal to eliminate LF as a public health problem by 2020 [1, 2] has made significant progress since its inception. About 7.1 billion cumulative treatments have been given towards interrupting transmission of LF through mass drug administration (MDA) of albendazole in combination with diethylcarbamazine (DEC) or ivermectin (2-drug regimen). At least 550 million people no longer require treatment out of about 1.3 billion people at risk of infection in 2000. As of 2017, 54 of the 72 LF endemic countries have fully implemented MDA, 13 countries have started MDA but have not up-scaled to all endemic districts and in the remaining 5 countries MDA is yet to be started [3]. WHO has formally acknowledged the claim of successfully eliminating LF as a public health problem in 11 countries (Cambodia, Cook Islands, Maldives, Niue, Sri Lanka, Togo, Vanuatu, Thailand, Egypt, Tonga and Marshall Islands) and in another 10 countries MDA has been stopped and post-MDA surveillance is in progress [4].

Transmission assessment survey (TAS) is the WHO-recommended protocol [5] to decide when to stop MDA (TAS 1) and to determine whether levels of infection have been sustained below the target threshold [antigen (Ag) prevalence <2% in 6 to 7-years-old children] after stopping MDA (‘post-MDA surveillance’). TAS is done in an evaluation unit (EU, an MDA implementation unit with a population not exceeding 2 million) if it has completed at least five rounds of MDA with an effective coverage (> 65%) and has recorded a microfilaria (Mf)-prevalence of < 1% or an Ag-prevalence of <2% in all the sentinel and spot-check sites. The sentinel and spot-check sites (geographical areas, each with a population of at least 500 people) are selected to collect parasitological data to monitor the impact of the MDA programme. While the sentinel sites remain the same, different spot-check sites are chosen for each assessment over the course of the MDA programme [5].

In India, if the assessment in all the sentinel and spot check sites indicates that the Mf-prevalence is below 1%, the Mf-prevalence is determined in 10 additional randomly selected sites to decide on conducting TAS [6]. In all 10 sites, Mf-prevalence should be below 1% for the area to conduct a TAS. TAS involves screening 6–7 year-old children through school or community-based surveys for detecting filarial antigen, as the children should have been protected from LF infection if MDA succeeded in interrupting transmission. When the Ag-prevalence in children is less than 2%, the EU is considered to have passed TAS and MDA will be stopped. If TAS has failed, MDA will continue.

The current recommendation for post-MDA surveillance is repeating TAS twice at 2–3 years (TAS 2) and 4–6 years (TAS 3) after stopping MDA. Evaluation unit that has cleared all the three TAS will be covered under post-validation surveillance for at least 5 years [4]. Post-MDA surveillance and mapping areas suspected to be LF endemic are required to prepare dossier for validation of LF elimination. However, factors related to sensitivity and evaluation unit size for TAS have been reported to limit its usefulness for confidently demonstrating transmission interruption in post-MDA phase [7–9]. A few countries including Sri Lanka [8], Tanzania [10], American Samoa [11, 12] and Ghana [13] have reported evidence of transmission despite passing TAS (< 2% Ag-prevalence in 6–7 year-old children) suggesting that TAS may be inadequate for taking the decision to stop MDA, when the infection in humans is at a very low level. Therefore there is an urgent need for more sensitive high-throughput and cost-effective diagnostic tools for detecting low infection signals during post-MDA situations [14].

India, the largest LF endemic country in the world (accounts for one-third of the global LF-burden [15] with 630 million people at risk of infection [16]), is one of the few countries that implemented MDA first for LF elimination following WHO guidelines [16]. The National Vector Borne Diseases Control Programme (NVBDCP) has implemented MDA with DEC alone from 2004, and co-administered DEC with albendazole from 2008 in all the 256 endemic districts (implementation units, IU) spread over 16 states and 5 union territories. At least 5 annual rounds of MDA with DEC plus albendazole have been completed in all the IUs. MDA was stopped in 100 IUs after passing TAS 1 (IU with more than 2 million population split into 2 or more EUs) and these IUs are currently under post-MDA surveillance; 25 IUs are being subjected to TAS 1 and in the remaining 131 IUs MDA is continuing. Currently the programme faces challenges as these IUs either failed TAS 1 (Ag-prevalence above the critical threshold of 2% for stopping MDA) or are not eligible for TAS as the infection levels remain above the critical threshold. The national programme has proposed to implement accelerated LF elimination strategy with MDA of a 3-drug regimen (DEC, albdendazole, ivermectin) to give a new impetus to the ongoing activities and achieve the ambitious goal of LF elimination by 2020 [16]. Since the effect of 3-drug regimen on adult worms is not yet fully understood, the currently recommended TAS in children based on antigen detection for 2-drug regimen may not be appropriate for deciding to stop or continue MDA with 3-drug regimen. Therefore, it is necessary to identify new target populations (human / mosquito), infection indicators, sampling strategies, and/or thresholds to determine when it is safe to stop MDA with 3-drug regimen.

Molecular xenomonitoring (MX, monitoring the presence of parasite DNA in mosquitoes) of infection in vectors has been recognized as a tool complementary to TAS and is used for monitoring recrudescence of infection in post-MDA [17, 18] and validation phases [19–21] when the infection is at a level lower than that detectable by Ag or Mf-testing. Vector infection of <0.25% for *Culex*, <1% for *Anopheles* and <0.1% for *Aedes* are suggested as provisional threshold levels to decide on stopping MDA [22]. MX involves collection of a large number of vector mosquitoes using appropriate collection methods, sampling techniques to obtain representative sample and detecting parasite DNA in pools of mosquitoes using PCR. Various collection methods have been employed and tested for their efficiency to capture different vector species: gravid traps [8, 19, 23–27], CDC light traps and battery powered aspirators for *Cx*. *quinquefasciatus* [28], AGT, a variant of OviART & pyrethrum spray method for *Anopheles gambiae* [13, 29, 30], and BG sentinel traps for *Aedes polynesiensis* [12]. MX technique is shown to be more efficient and sensitive in detecting parasite DNA than Mf-testing in humans [26, 31] and assessing the residual or renewed foci of infection after several rounds of MDA [8, 12, 19, 21, 26, 31–35]. LF endemic countries confirmed or supported the findings of TAS with MX on the basis of the absence of transmission during post-MDA or validation phases [19–21, 25]. Despite a number of studies which compared the decisions of TAS with those of MX, application of MX in operational settings requires an assessment of cost in relation to TAS and feasibility in terms of availability of adequate laboratory facilities and specially trained personnel [26].

In an earlier study, we developed and validated (i) a two-stage cluster design-based sampling strategy for collecting *Cx*. *quinquefasciatus* through gravid traps [19] and (ii) a cost-effective quantitative PCR assay for detecting *W*. *bancrofti* DNA in *Cx*. *quinquefasciatus* for MX at a sub-district level [36]. The present study aims to demonstrate the usefulness of the MX protocol at an evaluation unit level by comparing the results obtained from data for MX with those of microfilaremia in humans collected from the same communities, and also with the results of school-based TAS to provide a comprehensive assessment of the interruption of transmission. The usefulness and feasibility of MX as a complementary tool to TAS and for post-validation surveillance and its scope as a complementary tool for remapping areas suspected to be LF endemic for inclusion in the LF elimination programmes are discussed.

## Methodology

### Study area

This study was conducted between 2015–2016 in an IU in Cuddalore district (administrative unit), in Tamil Nadu, India, which is endemic for *Cx*. *quinquefasciatu*s transmitted *Wuchereria bancrofti*. This district has undergone 15 annual rounds (4 rounds of DEC alone and 11 rounds of DEC plus albendazole) of MDA during 1996–2014 with a break in 2005, 2006, 2008 and 2011. The programme reported MDA coverages (no. received the drug out of total population) were above 90% in all the rounds (S1 Table), whereas the coverage (no. received out of total no. of persons interviewed) by an independent assessment team in 2014 reported that it ranged from 70 to 83% (mean: 77%) with a consumption rate of 37–66% (mean: 54%, those swallowed the drug out of total no. of persons interviewed) in four randomly selected sites in the IU (S2 Table). As per national guidelines, pre-TAS assessment was done in two-stages. In stage 1, surveys were conducted in four sentinel and four spot-check sites. Mf prevalence was below 1% in all the 8 sites. In stage 2, Mf-survey was continued in 10 additional random sites (only in India). The Mf-prevalence was below 1% in each additional random site also, thereby the IU got qualified for TAS. The IU consists of seven sub-districts (Cuddalore, Chidambaram, Kurinchipadi, Panruti, Kattumannarkoil, Thittakudi, and Virudhachalam) having a total population of 2.6 million [37] with 1.7 million residing in rural areas (villages) and the rest in urban areas (wards). Since the total population is above 2 million, the IU was divided into two EUs as per the WHO protocol for conducting TAS [5]. Of the two EUs, one was selected randomly for the study.

**Fig 1:**
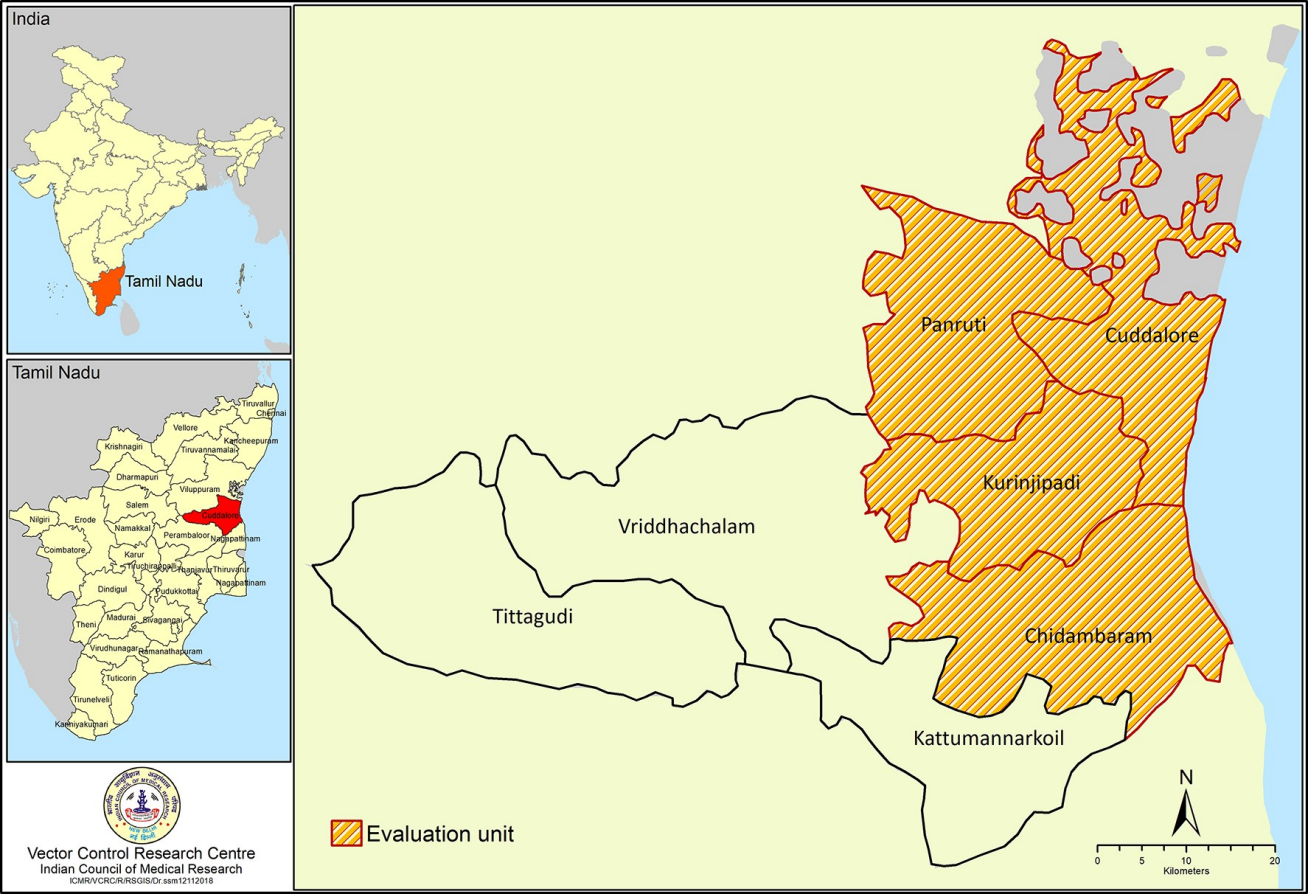
Map showing sub-districts of the studied evaluation unit (EU) in Cuddalore district, Tamil Nadu, India.

The selected EU has a population of 1.6 million and 397,468 households [37] administered by four sub-districts: (i) Cuddalore (population: 0.42 million), (ii) Chidambaram (population: 0.46 million), (iii) Kurinchipadi (population: 0.33 million) and (iv) Panrutti (population: 0.41 million) (Fig 1). Of these 58% live in rural villages and the remaining in urban wards.

### Vector sampling

**Fig 2:**
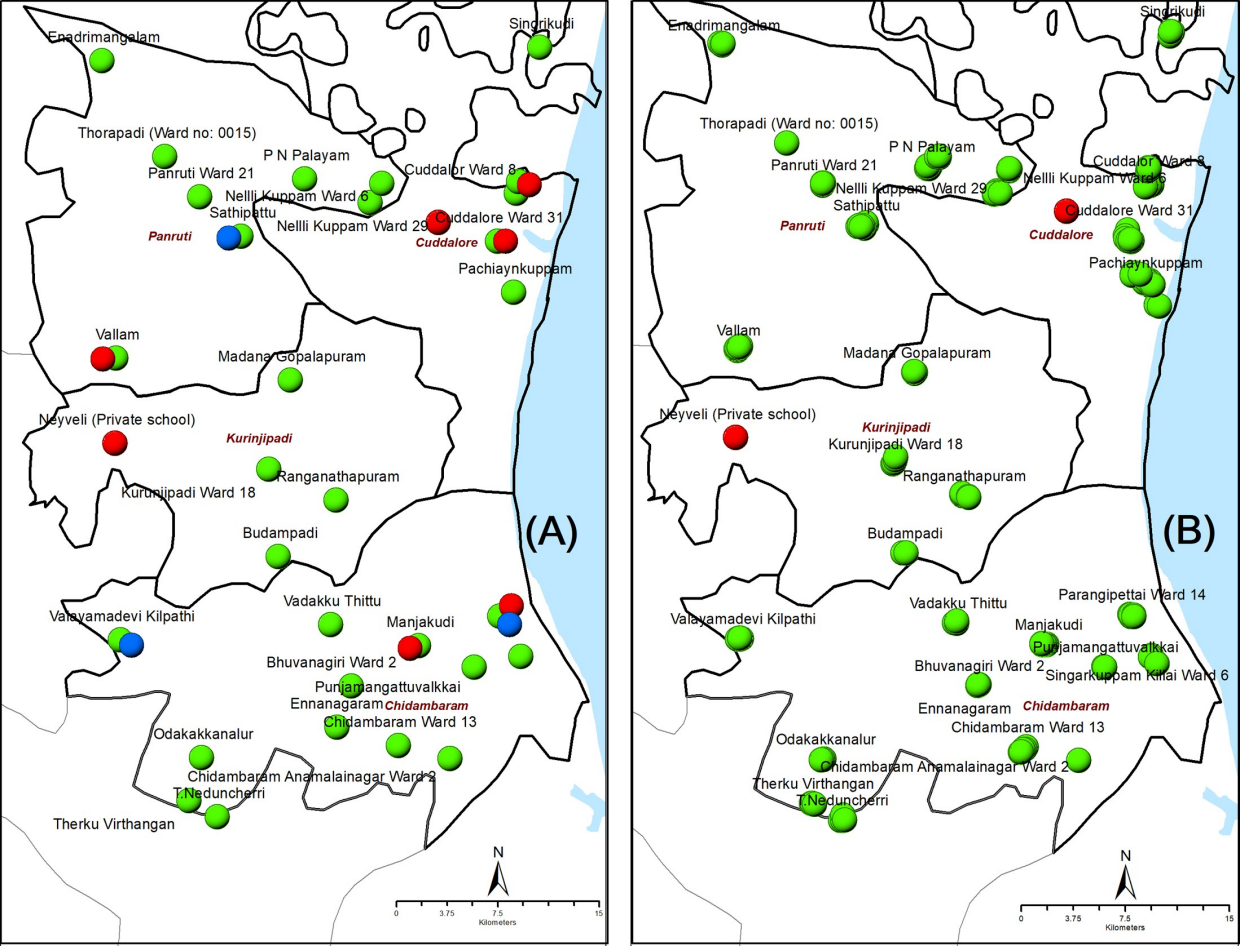
Location of clusters (villages or wards) for mosquito collection and human blood survey in the evaluation unit. The left panel shows the sites where mosquito and Mf-surveys were carried out (A) and the right-side panel shows the location of schools where the TAS was done (B). Clusters/schools that were negative for filarial infection in both humans and mosquitoes are shown in green, clusters with at least one mosquito pool positive for filarial infection or schools with at least one child positive for filarial antigen are shown in red and clusters with at least one Mf positive individual are shown in blue.

Vector surveys were carried out from January to August 2015 following a two-stage cluster sampling protocol, which was developed and validated at sub-district level for sampling *Cx*. *quinquefasciatu*s mosquitoes and molecular xenomonitoring (MX) of filarial infection. A detailed description of the sampling methodology is reported elsewhere [19]. In stage 1, 30 clusters (village or ward) were selected randomly from 669 clusters in all sub-districts of the EU (Fig 2A). In stage 2 (household, HH), on an average 5 HHs were selected per cluster with probability proportional to size (HHs) of the selected cluster. In each selected HH, gravid traps (a VCRC modified version of the CDC gravid trap, Model 1712, John W. Hock Co., Gainesville, FL), with hay infusion that attracts *Cx*. *quinquefasciatu*s [19], were placed outdoors within the household premises, at least 1 hour prior to sunset (1700 hrs) after obtaining oral consent from the residents. Geo-coordinates of all the mosquito collection points (HHs) were recorded using the Dell Axim X51 personal digital assistant (PDA) and mapped using ArcGIS (version 10.6, ESRI, Redlands, CA). The mosquito collection cages were removed from the traps the next morning (0630 hrs) and brought to the field laboratory. Mosquito collections in each selected HH were continued until a total of 50 *Cx*. *quinquefasciatu*s female mosquitoes (except unfed) were caught or for a maximum of 3–4 nights.

Collected mosquitoes were morphologically identified to species by experienced technical staff and female *Cx*. *quinquefascatus* further classified according to their abdominal appearance into gravid, semi-gravid, blood-fed and unfed. Unfed females were discarded; other ones were pooled by HH (25 or less mosquitoes/pool) in barcoded vials. Mosquito pools were dried at 95°C for a minimum of 15 minutes as described earlier [19] for later qPCR analysis. A maximum of 50 females (2 pools) were tested by HH. The target was to collect from all 30 clusters 7,500 females in 300 pools (30 clusters x 5 HHs per cluster x 25 mosquitoes per pool x 2 pools per HH = 7,500 mosquitoes).

### Filarial DNA extraction from mosquito pools

Filarial parasite DNA was extracted from each mosquito pool following indigenously developed simple TE (Tris-EDTA) -based DNA extraction procedure using bead beating (BB) for grinding the mosquitoes [38]. The DNA samples thus obtained were coded and analyzed by real-time quantitative PCR as described elsewhere [19, 36]. Briefly, each real-time PCR reaction was performed with 12.5 μl of FastStart Essential DNA probes Master (Roche Diagnostics, Germany) along with 450 nmol/L of each primer: LDR1-5’ATTTTGATCATCTGGGAACGTTAATA-3’; LDR2-5’CGACTGTCTAATCCATTCAGAGTGA-3’ and 125 nmol/L probe (6 FAM-ATCTGCCCATAGAAATAACTACGGTGGATCTG-TAMRA) (IDT, USA) in a final volume of 25μl in 96-well MicroAmp optical plates (Roche Diagnostics, Germany). One microliter of the extracted DNA was used as template in each real-time PCR reaction as described earlier [39]. Each plate (Lightcycler 480 Multi- well 96) was run with forty samples in duplicate. Three concentrations (1 ng, 100 pg and 10 pg) of DNA each in duplicate were used as positive control. Three negative control (only water and no template DNA) in duplicate were run simultaneously.

Real-time quantitative PCR reactions were carried out to determine the cycle of quantification (Cq) values for each sample. Thermal cycling parameters used were 50°C for 2min, 95°C for 10 min followed by 40 cycles of 95°C for 15 sec and 60°C for 1min. Thermal cycling and data analysis were done with Light Cycler 96 (Roche, Germany) instrument using sequence detection system (SDS) software (Applied Biosystems). Cq values of samples ranging from 1.0–39.0 were considered positive, and samples that failed to reach the fluorescence threshold beyond 39 were considered indeterminate and repeated to confirm the negativity or positivity of those samples as described by Rao et al. [39].

### Transmission Assessment Survey (TAS)

As required in the LF elimination programme, the TAS protocol described by WHO was used [5] to assess the impact for making decision on stopping MDA. List of schools with primary sections in the EU and the number of children enrolled in first and second grades in each school were obtained from the office of the district school education. Since the school enrolment rate in the EU was above 75%, school-based TAS was done considering the school (cluster) as a primary sampling unit, and children in grades I and II in the selected schools as secondary sampling unit. There were 887 primary schools with 36812 students in the first and second grades in this EU. The sample size and number of clusters for TAS was estimated using the survey sample builder (SSB) with an estimated non-response rate of 10%. As per SSB, a sample of 1556 children from 43 systematically selected clusters (Fig 2B) were chosen and targeted with a critical cut-off of 18 positives for deciding to stop MDA. All the selected children were tested for filarial antigenemia using the immonochromatographic card test (ICT) manufactured by BinaxNow (Scarborough, USA) [40] during the period from 26 March 2015 to 9 April 2015. From each child 100μl blood was collected and loaded directly on to the ICT cards. A team of 4 trained technicians conducted the survey and the results of the test were read at 10 minutes’ interval and positives were recorded.

### Community surveys for microfilaraemia

Night blood survey was carried out from October 2016 to January 2017 to assess the prevalence of microfilaria (Mf) in each of the 30 sites where xenomonitoring was done (Fig 2A). All available and consenting persons, above 5 years old, in each systematically selected house were tested for Mf with 60μl thick blood smears prepared with blood collected by finger prick method between 1930 hrs and 2330 hrs, which was operationally feasible. Sample size (6085) was calculated for an expected Mf-prevalence of 1% (based on sentinel, spot-check and additional 10 random sites, S3 Table) with 0.25% precision and 95% CI. The calculated sample size after adjusting for a design effect of 1.5 was ~ 9200 persons. Considering a family size of four persons, 2300 houses were selected randomly with probability proportion to population size of the respective village. Blood slides were dehaemoglobinized with distilled water, dried, fixed in acidified methyl alcohol and stained with JSB-1 stain. Trained microscopists examined the blood slides and the number of Mf in each positive slide was recorded. All the positive slides and 5% of the negative slides selected randomly were cross-examined for quality assurance.

### Data analysis

Mosquito ‘pool infection rate’ was calculated as the percentage of pools positive for *W*. *bancrofti* DNA relative to the number of pools screened by individual cluster, sub-district and for the EU as a whole. The prevalence of *W*. *bancrofti DNA* (maximum likelihood estimates with 95% confidence interval) was estimated with PoolScreen software 2.0.3 [41, 42]. Mf-prevalence in human was calculated as the ratio of number of blood slides positive for Mf to total number of slides examined. Kruskal-Wallis one-way analysis of variance was used to compare the difference in the density (number per trap-night) of *Cx*. *quinquefasciatu*s (gravid, semi-gravid and freshy-fed) among sub-districts. Random effects logistic regression model was used to compare the differences across sub-districts in terms of (i) prevalence of Mf in human and (ii) percentage of *Cx*. *quinquefasciatu*s: *(a)* captured by gravid traps out of all mosquito species, (b) with various abdominal status (blood-fed, semi-gravid, gravid, except unfed), and (c) pools positive for *W*. *bancrofti* DNA. Separate analysis was done for each of the above-mentioned parameters (response variable) with sub-districts and sites as fixed and random effects respectively. P values <0.05 were considered statistically significant.

### Costing

Costs incurred in conducting MX and TAS were estimated per EU using itemized cost menu. For MX, the cost components include (i) personnel engaged for mosquito collection, preservation and processing, (ii) transport for the field surveys, (iii) field supplies (traps, vials, and barcodes) and (iv) lab processing of samples (reagents and primers).

The cost components for TAS include (i) personnel, (ii) transport to the field sites, and (iii) supplies (ICT, Binax, USA) and field supplies (lancets, cotton rolls, and surgical spirit). TAS was conducted jointly by the research team and the district health system. Therefore, the services of the health staff were included as ‘opportunity cost’ (the cost for diverted services by which the benefit of other services is lost) under ‘personnel’ head. In the present study, we have used the ICT kits from the national programme, donated by the WHO Regional Office in the country. Therefore, the cost of ICT was included as ‘opportunity cost’. Further, since the national programme currently uses filariasis test strip (FTS, Alere, Scarborough, ME) instead of ICT [40, 43], costing of TAS with FTS was also done for the purpose of comparison.

### Ethics statement

The Institutional Human Ethics Committee of the ICMR-Vector Control Research Centre approved the study proposal. Survey teams explained the purpose of the study and study procedures to the selected household members. Written informed consent was obtained from all consenting adults and parents for children for collecting finger-prick blood to assess Mf and filarial Ag. Personal identity and results of tests were kept confidential. A list of persons detected positive for Mf or Ag was shared with the Deputy Director of Health Services of the District with a request to treat filarial infected persons as per the national programme guidelines of India. Since gravid traps for mosquito collection were placed outside the households and it did not interfere with any domestic activities within or around the households, the xenomonitoring part of the study does not involve any ethical issue, but oral consent was obtained from the head of the households.

## Results

### Sampling *Cx*. *quinquefasciatus* mosquitoes

**Fig 3:**
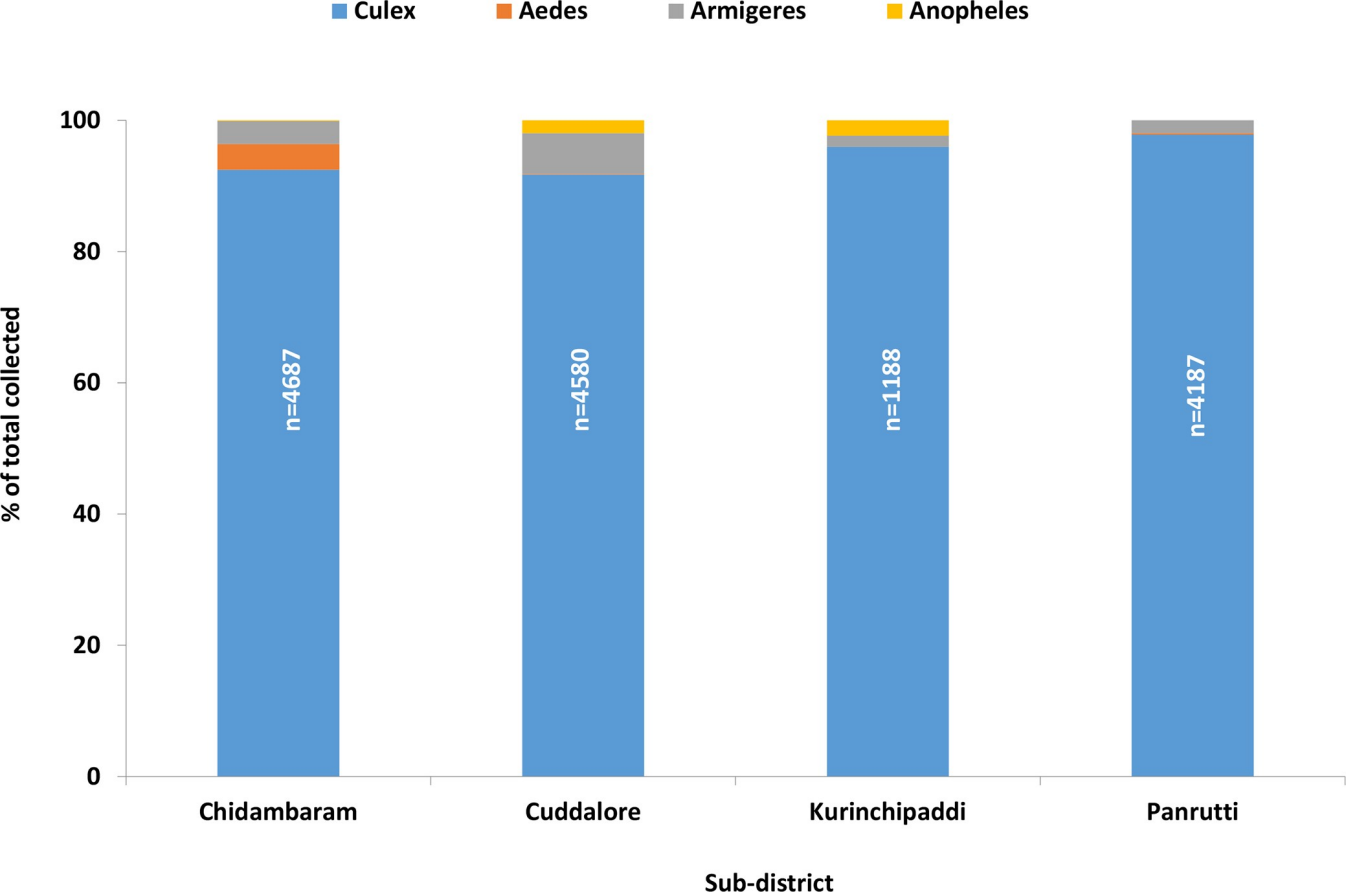
Mosquito species composition in gravid trap collection by sub-districts in the evaluation unit.

**Fig 4:**
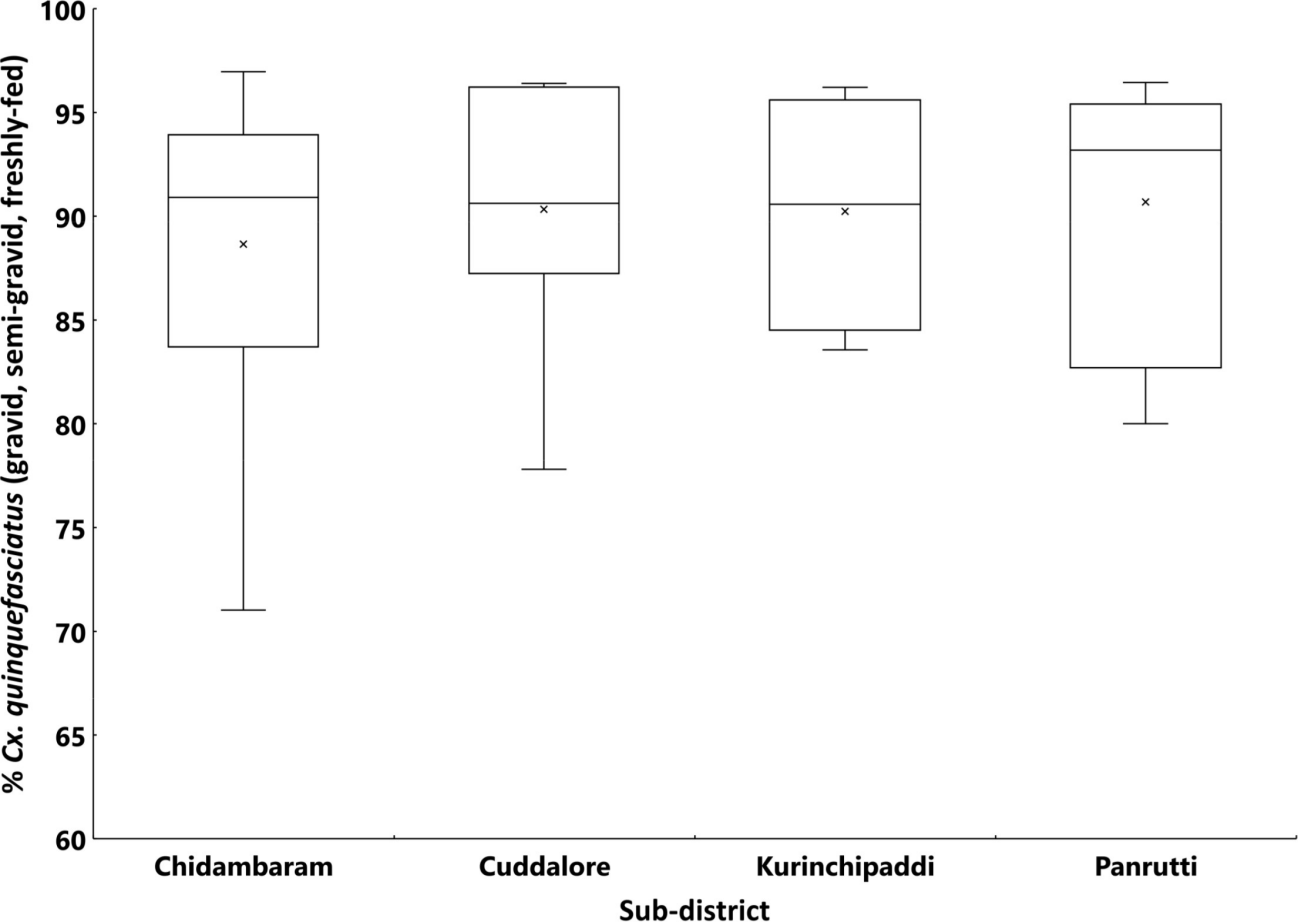
Box-plot showing the percentage *Culex quinquefasciatus* female mosquitoes (gravid and blood-fed, except unfed) among the total collected by gravid traps in different clusters by sub-district in the evaluation unit. The horizontal line inside the box shows the median and the 'x' is the mean. The ends of the box are the 25th and 75th percentiles of the distribution of the percentage of mosquitoes. The whiskers extend to 1.5 times the height of the box (i.e. the interquartile range, IQR). If the data are distributed normally, approximately 95% of the data are expected to lie between the inner fences.

**Fig 5:**
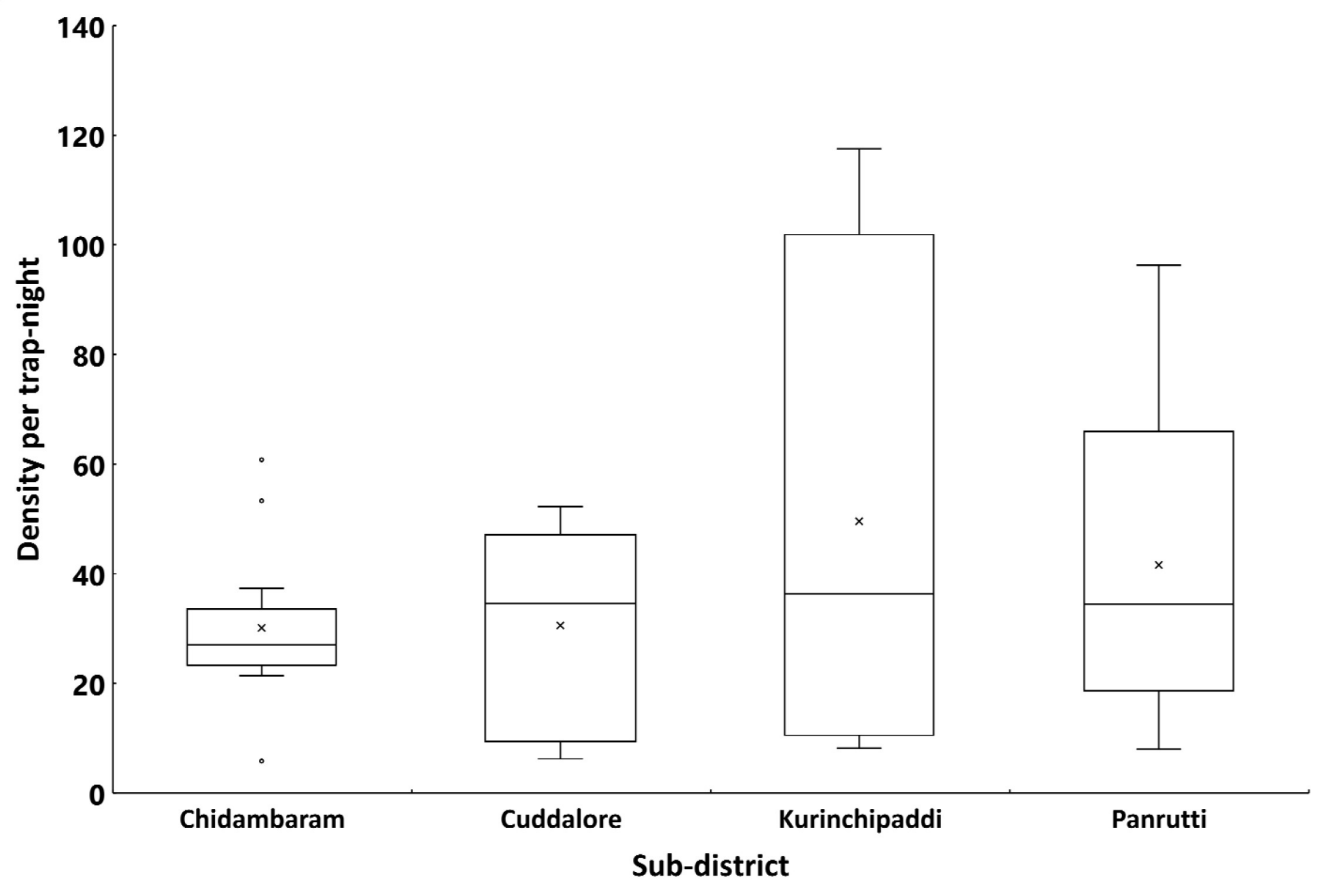
Density (Numbers of females/trap-night) of *Culex quinquefasciatus* female mosquitoes (gravid and blood-fed, except unfed) in different clusters by sub-districts in the evaluation unit. The horizontal line inside the box shows the median and the 'x' is the mean. The ends of the box are the 25th and 75th percentiles of the distribution of the density. The whiskers extend to 1.5 times the height of the box (i.e. the interquartile range, IQR). If the data are distributed normally, approximately 95% of the data are expected to lie between the inner fences. Values above or below the upper or lower whiskers are labelled as outliers (o).

A total of 14,642 female mosquitoes comprising four species were collected spending 407 trap nights in 185 households spread over 30 clusters in the EU. Fig 3 presents the percentage composition of each species by sub-district. The filariasis vector, *Cx quinquefasciatus*, was the predominant species (94.1%), followed by *Armigeres* (3.8%), *Aedes* (1.4%) and *Anopheles* (0.8%). A similar pattern was observed in all the four sub-districts (>90% *Cx*. *quinquefasciatu*s, Fig 3). Of the 13771 *Cx*. *quinquefasciatu*s females collected in the EU, more than 90% were gravid, semi-gravid or freshly-fed in all the sub-districts (range: 71–97% in different clusters, Fig 4). The mean density of *Cx*. *quinquefasciatu*s mosquitoes (gravid, semi-gravid and freshly-fed) in the EU was 30.8 per trap-night (range: 5.8–117.5 per trap-night in different clusters). The density averaged for the sub-districts ranged between 30.1 and 49.6 per trap-night (Fig 5). The sub-districts did not differ significantly in terms of percentage of *Cx*. *quinquefasciatu*s among all species captured by gravid trap (P = 0.06), its percentage other than unfed (P = 0.32), and the density per trap-night (P = 0.95).

### Vector infection rate

A total of 358 pools were formed out of 8850 gravid *Cx*. *quinquefasciatu*s collected from 185 HHs (as against 150 HHs targeted) spread over 30 clusters in the EU. As many as 353 (98.6%) pools had a pool size of above 20 each as against the desired pool size of 25 [22]. Two mosquito pools were screened from each of 173 HHs (pools of 25 mosquitoes each were from 166 HHs, and of 5–25, median 24 females were from seven HHs). One mosquito pool (16–25, median 22, females) was screened from each of remaining 12 HHs. The qPCR (quantitative PCR) results by cluster and sub-district are summarized in Table 1. Of 358 pools tested, 11 (3.1%) pools from five clusters found positive for *W*. *bancrofti* DNA, representing three of the four sub-districts (range: 2.0 to 4.7%) in the EU. It is interesting to note that none of the 26 pools were screened positive in Kurinjipadi sub-district. Statistical analysis revealed that the pool infection rates did not differ significantly among sub-districts (P = 0.56).

**Table 1:** PoolScreen infection of *Culex quinquefasciatus* mosquitoes with *Wuchereria bancrofti* DNA in the evaluation unit during January-August 2015. Clusters with one or more positive pools are shaded in orange.

Cluster No.	Sub-district	Cluster	No. of houses sampled	No. of *Cx*. *quinquefasciatu*s collected	No. of pools screened	No. of pools infected	Pool infection rate (%)	*W*. *bancrofti* DNA prevalence (95% CI)
1	Chidambaram	Bhuvanagiri Ward 2	3	150	6	0	0.00	0.0 (0.0–2.0)
2		Chidambaram Anamalainagar Ward 2	1	50	2	0	0.00	0.0 (0.0–4.2)
3		Chidambaram Ward 13	5	217	9	0	0.00	0.0 (0.0–1.5)
4		Ennanagram	2	100	4	0	0.00	0.0 (0.0–2.7)
5		Manjakudi	12	541	22	1	4.55	0.18 (0.01–0.90)
6		Odakakkanalur	3	150	6	0	0.00	0.0 (0.0–2.0)
7		Parangipettai Ward 14	5	250	10	4	40.00	1.9 (0.63–4.70)
8		Punjamangattuvalkkai	2	36	2	0	0.00	0.0 (0.0–5.8)
9		Singarkuppam Killai Ward 6	4	200	8	0	0.00	0.0 (0.0–1.6)
10		T.Neduncherri	4	197	8	0	0.00	0.0 (0.0–1.6)
11		Therku Virthangan	3	150	6	0	0.00	0.0 (0.0–2.0)
12		Vadakku Thittu	4	200	8	0	0.00	0.0 (0.0–1.6)
13		Velayamadevi Kilpathi	8	390	16	0	0.00	0.0 (0.0–0.9)
** **	**Chidambaram Sub-district**		**56**	**2631**	**107**	**5**	4.67	**0.20 (0.07–0.45)**
14	Cuddalore	Cuddalore Ward 31	9	450	18	3	16.67	0.71 (0.19–1.90)
15		Cuddalore Ward 8	13	623	26	0	0.00	0.0 (0.0–0.6)
16		Nellikuppam Ward 29	5	250	10	0	0.00	0.0 (0.0–1.3)
17		Nellikuppam Ward 6	5	250	10	0	0.00	0.0 (0.0–1.3)
18		Pachia Kuppam	21	1050	42	0	0.00	0.0 (0.0–0.3)
19		Peria Kangankuppam	5	250	10	1	10.00	0.40 (0.02–2.00)
20		Singrikudi	5	250	10	0	0.00	0.0 (0.0–1.3)
** **	**Cuddalore Sub-district**		**63**	**3123**	**126**	**4**	3.17	**0.13 (0.04–0.31)**
21	Kurinjipadi	Buddampadi	2	75	3	0	0.00	0.0 (0.0–3.2)
22		Kurunjipadi Ward 18	4	200	8	0	0.00	0.0 (0.0–1.6)
23		Madana Gopalapuram	4	200	8	0	0.00	0.0 (0.0–1.6)
24		Ranganathapuram	5	171	7	0	0.00	0.0 (0.0–1.8)
** **	**Kurinjipadi Sub-district**		**15**	**646**	**26**	**0**	0.00	0.0 (0.0–0.6)
25	Panrutti	Enadrimangalam	5	224	9	0	0.00	0.0 (0.0–1.4)
26		P N Palayam	13	650	26	0	0.00	0.0 (0.0–0.5)
27		Sathipattu	13	650	26	0	0.00	0.0 (0.0–0.5)
28		Thorapadi	1	23	1	0	0.00	0.0 (0.0–6.6)
29		Vallam	13	650	26	2	7.69	0.31 (0.06–1.00)
30		Panruti Ward 21	6	253	11	0	0.00	0.0 (0.0–1.3)
	**Panrutti Sub-district**		**51**	**2450**	**99**	**2**	2.02	**0.08 (0.01–0.27)**
	**Overall for Evaluation Unit**		**185**	**8850**	**358**	**11**	3.07	**0.13 (0.07–0.22)**

Pool Screen estimate revealed that the prevalence of *W*. *bancrofti* DNA for the EU (overall sub-districts) was 0.13% (95% CI: 0.07–0.22%) (Table 1). This rate is much lower than the suggested critical cut-off of 0.25% [22, 31, 44] for transmission to take place at sub-district and EU level. The parasite DNA rates as estimated by PoolScreen vary greatly among clusters (range: 0.0–1.9%). In four of the 30 clusters, the prevalence of filarial DNA in mosquitoes exceeded the provisional target of 0.25%. Moreover, surprisingly the estimated upper 95% CI for the prevalence of *W*. *bancrofti* DNA exceeded the provisional target in all the 30 clusters (Table 1).

### Transmission assessment survey (TAS)

Table 2 shows the results of TAS by school and sub-district. A total of 1579 (including one invalid ICT test) children in grades I and II were tested from 43 schools selected for TAS. The actual number of children screened exceeded the target sample size of 1556. The excess sampling could be due to a higher non-response rate (10%) assumed in the calculation of sample size using SSB than the actual. Among the remaining 1578 children screened, only four (0.26%) were Ag positive by ICT and were located in two sub-districts. Three out of 34 children in one school and one out of 77 children in another school were Ag-positive. In one school the Ag-prevalence exceeded the critical level of 2%. Ag-prevalence ranged between 0.0 and 0.6% in the sub-districts. Since the number of Ag positive children is less than the critical cut-off (18), the EU passed TAS and qualified for stopping MDA. The village or ward in which schools selected for TAS were located were different from the clusters (villages or wards) selected for MX and Mf-surveys and hence there was no parallel data for comparison.

**Table 2:** Results of transmission assessment survey (TAS) in the selected schools in the evaluation unit conducted during April 2015. Schools with one or more filarial antigen positive children are shaded in orange.

School ID	Sub-district	Primary Health Centre	Health Sub-Centre	Location (government / Private School)	No. Blood Samples		ICT +ve	
1	Chidambaram	Bhuvanagiri	Keelbhuvanagiri	Keezhbhuvanagiri (Govt.)	12		0	
2		Kavarapattu	C.Kothangudi	C.Kothangudi (Govt.)	22		0	
3		Kavarapattu	C.Kothangudi	C.Kothangudi (Govt.)	5		0	
4		Kavarapattu	Kavarapattu	Kavarapattu (Govt.)	35		0	
5		Kavarapattu	Uthamasolamangalam	Sithalapadi (Govt.)	8		0	
6		Krishnapuram	Manjakollai	Manjakollai (Govt.)	32		0	
7		Orathur	Mathurandaganallur	Devangudi (Private)	13		0	
8		Orathur	Orathur	Paradhur Chavadi (Govt.)	9		0	
9		Orathur	Sathamangalam	Vadapakkam (Govt.)	5		0	
10		Orathur	Sathamangalam	Vaiyalur (Govt)	9		0	
11		Palayankottai	Nangudi	Vizhuperundhurai (Govt.)	12		0	
12		Palayankottai	Ramapuram	T. Viruthangam (Govt.)	24		0	
13		Palayankottai	Ramapuram	Therkupalayam (Govt.)	31		0	
14		Puduchattram	C.Pudupettai	Parangipettai (Private)	101		0	
15		Puduchattram	Periyapattu	Periyandikuzhi (Govt.)	11		0	
16		Sethiathoppu	Melvalayamadevi	Kathazhai (Govt.)	24		0	
17		Sethiathoppu	Nellikollai	Thurinjikollai (Private)	14		0	
18		Vallampadugai	Vallampadugai	Vallampadugai (Private)	81		0	
19		Vilagam	Thunisiramedu	Mugaiyur (Govt.)	25		0	
	**Chidambaram Sub-district**					**473**		**0**	
20	Cuddalore	Cuddalore Municipality	Pudhupalayam	Pudhupalayam (Private)	63		0	
21		Cuddalore Municipality	Cuddalore OT	Cuddalore OT (Private)	256		0	
22		Cuddalore Municipality	Manjakuppam	Manjakuppam (Govt)	13		0	
23		Cuddalore Municipality	Thirupapuliyur	Thirupapadhiriyur (Govt.)	33		0	
24		Karaikadu	Karaikadu	Kannarapettai (Govt.)	21		0	
25		Karaikadu	Pachyankuppam	Vazhisodhipalayam (Govt.)	17		0	
26		Nellikuppam Municipality	Nellikuppam	Nellikuppam (Govt)	12		0	
27		Oraiyur	Aviyanur	Aviyanur (Govt.)	23		0	
28		Oraiyur	Chinnapettai	Chinnapettai (Govt.)	26		0	
29		Thiruvanthipuram	Thiruvanthipuram	Thiruvanthipuram (Govt.)	34		3	
30		Thukkanampakkam	Kalaiyur	Erandayiramvilagam (Govt.)	15		0	
	**Cuddalore Sub-district**					**513**		**3**	
31	Kurinjipadi	Neyveli Lignite Corporation	Neyveli Lignite Corporation	Neyveli (Private)	77		1	
32		Puliyurkattusalai	Mathanagopalapuram	Peikanatham (Govt.)	12		0	
33		Puliyurkattusalai	Puliyurkattusalai	Puliyurkattsagai (Govt.)	9		0	
34		Thiruchopuram	Andarmullipallam	Andarmullipallam (Govt.)	34		0	
35		Thiruchopuram	Andarmullipallam	Thorapadi (Govt.)	52		0	
36		Thondamanatham	Sammattikuppam	Sammitikuppam (Govt.)	12		0	
37		Vadalur	Karunkuli	Kollakurdi(Govt)	11		0	
	** Kurinjipadi Sub-district**					**207**		**1**	
38	Panrutti	Marungur	Marungur	Kollukarankuttai Vallalar (Private)	281		0	
39		Panruti Municipality	Panruti	Tharkavandimedu (Govt.)	4		0	
40		Perperiyankuppam	Keelkangeyankuppm	Athirikuppam (Govt.)	13		0	
41		Perperiyankuppam	PP Kuppam	Muthandikuppam (Govt.)	16		0	
42		VP Nallur	Thiruvamoor	Kolapakkam (Govt.)	7		0	
43		VP Nallur	Thiruvamoor	Thiruvamur (Govt.)	65		0	
	**Panrutti Sub-district**					**386**		**0**	
	**Overall for Evaluation Unit**					**1579**		**4**	

Govt.—Government School

Private–Private School

### Mf-prevalence

As many as 9804 persons (against 9200 targeted) from 2981 households (15.5% of 19179 HHs) located in the 30 randomly selected clusters were screened for Mf. The percentage of individuals sampled varied between 12 and 21% among clusters (Table 3). In the evaluation unit selected, only four individuals from three clusters of two sub-districts were screened positive for Mf (0.04%; 95% CI: 0.01–0.1%) with Mf counts ranging from 2 to 16 (mean: 8 per 60μl blood). Mf-prevalence in all the three clusters were less than 1%, indicating that the prevalence in the EU is below the critical threshold (<1%) for transmission to continue. Though the observed Mf-prevalence was below the critical threshold in all 30 clusters, their upper 95% confidence limits exceeded the critical level in 24 of these clusters. Mf-prevalence did not differ significantly (P = 0.81) between sub-districts (range: 0.0% to 0.07%).

**Table 3:** Microfiilaria (Mf) prevalence in clusters from which *Culex quinquefasciatu*s mosquitoes were sampled during October 2016-January 2017. Clusters with one or more positive individuals are shaded in orange.

Cluster No.	Sub-district	Site Name	Population	No. of blood samples Screened	Fraction Sampled (%)	Mf-prevalence (95% CI) (Clopper-Pearson interval)
1	Chidambaram	Bhuvanagiri Ward 2	1152	142	12.3	0 (0.0–2.6)
2		Chidambaram Anamalai Nagar Ward 2	542	113	20.8	0 (0.0–3.2)
3		Chidambaram Ward 13	2188	272	12.4	0 (0.0–1.3)
4		Ennanagram	774	120	15.5	0 (0.0–3.0)
5		Manjakudi	5949	745	12.5	0 (0.0–0.4)
6		Odakakkanalur	1092	145	13.3	0 (0.0–2.5)
7		Parangipettai Ward 14	2271	275	12.1	0.4 (0.1–2.0)
8		Punjamangattuvalkkai	520	74	14.2	0.0 (0.0–4.8)
9		Singarkuppam Killai Ward 6	1769	227	12.8	0.0 (0.0–1.6)
10		T.Neduncherri	1817	240	13.2	0.0 (0.0–1.5)
11		Therku Virthangan	1054	144	13.7	0.0 (0.0–2.5)
12		Vadakku Thittu	1738	220	12.7	0.0 (0.0–1.7)
13		Velayamadevi Kilpathi	3598	447	12.4	0.2 (0.01–1.2)
** **	**Chidambaram Sub-district**		**24464**	**3164**	12.9	**0.06 (0.01–0.2)**
14	Cuddalore	Cuddalore Ward 31	3345	415	12.4	0.0 (0.0–0.9)
15		Cuddalore Ward 8	4543	547	12.0	0.0 (0.0–0.7)
16		Nellikuppam Ward 29	1700	259	15.2	0.0 (0.0–1.4)
17		Nellikuppam Ward 6	1722	220	12.8	0.0 (0.0–1.7)
18		Pachia Kuppam	7857	932	11.9	0.0 (0.0–0.4)
19		Paria Kangankuppam	1543	204	13.2	0.0 (0.0–1.8)
20		Singrikudi	1703	233	13.7	0.0 (0.0–1.6)
	**Cuddalore Sub-district**		**22413**	**2810**	12.5	**0.0 (0.0–0.13)**
21	Kurinjipadi	Buddampadi	973	127	13.1	0.0 (0.0–2.8)
22		Kurunjipadi Ward 18	1537	196	12.8	0.0 (0.0–1.9)
23		Madana Gopalapuram	2063	277	13.4	0.0 (0.0–1.3)
24		Ranganathapuram	2529	299	11.8	0.0 (0.0–1.2)
	**Kurinjipadi Sub-district**		**7102**	**899**	12.7	**0.0 (0.0–0.4)**
25	Panrutti	Enadrimangalam	2293	281	12.3	0.0 (0.0–1.3)
26		P N Palayam	7317	875	12.0	0.0 (0.0–0.4)
27		Sathipattu	6018	714	11.9	0.3 (0.03–1.0)
28		Thorapadi	293	41	14.0	0.0 (0.0–8.6)
29		Vallam	6309	753	11.9	0.0 (0.0–0.5)
30		Panruti Ward 21	2002	267	13.3	0.0 (0.0–1.4)
	**Panrutti sub-district**		**14622**	**2931**	20.0	**0.07 (0.01–0.2)**
	**Overall for Evaluation Unit**		68601	9804	12.6	**0.04 (0.01–0.10)**

A parallel comparison of the Mf-prevalence in these clusters with the prevalence of parasite DNA estimated by xenomonitoring showed that the prevalence estimates are comparable between the two survey methods (human vs mosquito) in one of the three clusters (95% CIs for Mf-prevalence in human and parasite DNA prevalence in vector overlap with each other).

### Cost of surveys

Table 4 compares the costs of school-based TAS and MX survey for assessing LF transmission in the EU. The total cost for conducting MX at an EU level was estimated to be $14,259 USD (1USD = Rs.73.72) per EU. The major cost component was personnel, accounting for 64.6% of the total cost, followed by transportation (17.1%). The total cost for conducting TAS with ICT in the EU was estimated to be $14,103.8 USD and the cost per school was $328 USD, and cost per child was $9.06 USD. The major cost component for TAS was ‘supplies’, accounting for about 60.5% of the total cost, followed by ‘personnel’ (37.5%) and transportation (2%); ICT alone shares about 97.7% ($8443 USD) of the total cost of supplies (Table 4). The total cost of xenomonitoring is more than the school-based TAS using ICT by $155.2 USD.

**Table 4:** Costs (US $) of transmission assessment survey (TAS) and molecular xenomonitoring (MX) for assessing lymphatic filariasis transmission in the evaluation unit.

Heads	Cost of school-based TAS with ICT (%)	Cost of MX (%)
**Personnel**	**5286.5 (37.5)**	**9070.5 (63.6)**
**Transport**	**287.7 (2.0)**	**2442.9 (17.1)**
**Supplies**		
ICT	8334.0 (59.1)	NA
Field supplies	195.6 (1.4)	1657.6 (11.6)
**Sub-total (Supplies)**	**8529.6 (60.5)**	**1657.6 (11.6)**
**Lab processing**	**NA**	**1088.0 (7.6)**
**Total cost**	**14,103.8**	**14,259.0**

NA—Not applicable

## Discussion

This is the first MX evaluation study carried out at an EU level in India to monitor *W*. *bancrofti* infection in *Cx*. *quinquefasciatus* mosquitoes. The study compares the results of MX with those of human Mf-survey carried out via community-based sampling (not WHO protocol) of 30 clusters (villages or wards), and TAS via ICT and conducted through a school-based sampling of 43 schools in the EU. After 15 rounds of MDA, when mosquito infection levels are expected to be very low, gravid traps yielded enough mosquitoes for transmission assessment by MX. Further, MX implemented in 2015 revealed that the prevalence of *W*. *bancrofti* DNA in *Cx*. *quinquefasciatus* was below the 0.25% provisional critical threshold [22, 31, 44], suggesting interruption of transmission in the EU. The TAS in 2015 showed that the rate of antigen-positive children was below the 2% critical threshold, indicating as well interruption of transmission and that the EU is qualified for stopping MDA. Our data therefore validated MX as a complementary tool to stop MDA in the EU. One year later (2016), a blood survey for microfilaremia showed that “none of the clusters was above the 1% Mf-threshold”, thus confirming the results of MX and TAS.

### MX and TAS for post-MDA surveillance and validation

Several countries with different epidemiological settings applied MX to generate evidence for absence of active transmission during post-MDA situation. Many of these studies reported that the prevalence of *W*. *bancrofti* DNA in *An*. *gambiae* [in Sierra Leone, Togo 20, 33, 45] or in *Cx. quinquefasciatus [in Bangladesh and India, 19, 21, 46]* are well below the provisional threshold for transmission interruption [1% and 0.25% for *An*. *gambiae* and *Cx*. *quinquefasciatus* respectively, 22]. The MX results in all the above studies corroborated with TAS findings (Ag-prevalence below the threshold of 2% among children) that led to stopping MDA in endemic districts after multiple rounds of MDA. However, a few studies in Sri Lanka [8, 26, 47] and in American Samoa [9, 12, 34] have shown that the prevalence of *W*. *bancrofti* DNA in *Cx*. *quinquefasciatus* (>0.25%) and *Aedes polynesiensis* (>0.1%) were above the provisional transmission thresholds for the respective vector-parasite combination, despite the Ag-prevalence by TAS in the EUs were below the critical threshold of 2%. In American Samoa, school-based third TAS and the community-based serological studies carried out 10 years after cessation of MDA confirmed recrudescence of LF transmission, as indicated by the MX before third TAS [9, 34].

Derua et al. [35] have reported that the prevalence of *W*. *bancrofti* DNA in *An*. *gambiae* complex and *An*. *funestus* group in Mafia islands, Tanzania were above the provisional thresholds (vector infection rate of 1.7%), where the antigen positivity (4%) among 6–9 year-old children was also above the threshold level, despite nine rounds of MDA.

Application of MX, prior to MDA in Conakry, Guinea, in West Africa revealed that the prevalence of *W*. *bancrofti* DNA by LAMP (loop-mediated isothermal amplification assay) in both *An. gambiae* and *Cx*. *quinquefasciatu*s were above the provisional threshold for interruption of transmission, despite the absence of circulating filarial antigen in human population [32]. Based on the above findings, we conclude that MX can be used as a complement to TAS for stopping MDA, detecting residual foci of infection during post-MDA or post-validation phases and for mapping areas to initiate MDA.

### MX for assessing residual hotspots

WHO, while recommending MX for post-MDA surveillance, suggested focussing on mosquito surveys on individual villages to provide an indication of the presence of residual infection (residual hotspots) [46] at village level [18]. Rao et al. [48], after analysing MX data by sites reported persistent infection in many sites in an EU in Sri Lanka that stopped MDA post-5 rounds after demonstrating transmission interruption through TAS. Our MX survey detected 5 clusters with residual infection compared to 3 clusters detected by community-based Mf-survey or 2 schools by Ag-survey through TAS, suggesting that MX was more sensitive than community-based Mf-survey or school-based Ag-survey by TAS for detecting residual infection in areas under post-MDA surveillance.

In all the 30 clusters, the upper 95% CI for *W*. *bancrofti* DNA prevalence exceeded the provisional vector infection threshold of 0.25% (Table 1). Considering only the cluster wise point estimates, the results of MX reveal that 13.3% (4/30 clusters; 95% CI: 3.8–30.7%) of the clusters in the EU are expected to exceed the suggested critical threshold of 0.25% infection in vector for transmission interruption. This means that on an average one would expect 89 of the 669 clusters in the EU to be under the risk of resurgence. Further, among the five clusters positive by MX, one cluster was also positive for Mf in humans. A post-hoc power analysis indicated that the sample size for this cluster is adequately powered (>90%) to reject the null hypothesis that the prevalence of infection in mosquitoes is <0.25%, the threshold for transmission interruption. This suggests that about 3.3% (1 out of 30 clusters sampled) of the 669 clusters (i.e. 20 clusters) in the EU are expected to have residual human Mf carriers and therefore are at risk of transmission. Our site wise analysis of the MX data suggests that MX could be a highly sensitive tool for detecting residual hotspots during post-MDA surveillance phase.

It is important that the residual hotspots in an EU are monitored and appropriate site-specific interventions, such as vector control or screening and treating individuals for filarial infection initiated to prevent resurgence of infection during post-MDA phase. Since MX will detect only those hotspots within the selected clusters, the presence of other such hotspots in the EU can be identified using the environmental risk factors as proxy indictors, as reported in a recent study in India [49].

However, it is important to note that the samples for MX, Mf and TAS are powered only to take a decision at an EU level and not at a cluster level. We have drawn inference about transmission or residual infection status at the cluster level (village, school) based on the estimated prevalence of infection (Mf and Ag) for individual clusters. Similar assessment has been made by others [8, 9, 26, 33, 48]. The individual cluster level estimate (for MX or TAS) could only give an indication, considering the small sample size for the individual clusters. Further studies with appropriate sample size for each cluster are warranted to provide quantitative assessment of transmission levels or residual infection. Such an estimate could be used as an additional criterion to supplement the TAS decision for stopping MDA in an EU, as had been recommended as ‘dual thresholds’ for stopping MDA in onchocerciasis control programmes in Africa [50].

### Sampling: MX, Mf and TAS

Our data from both MX and Mf-surveys confirmed the presence of residual infection in a few of the communities in the EU, MX being more sensitive in detecting *W*. *bancrofti* persistence than the Mf-survey in humans (5 clusters positive by MX vs 3 clusters by Mf in human). MX detected filarial DNA in clusters where no infection was detected in humans by Mf-survey. Similar observation has also been reported in Sri Lanka [47]. This could be due to a higher efficiency of mosquitoes in picking up infection as reported earlier as a phenomenon of “limitation” [51–53]. However, MX did not show vector infection in two of the three clusters positive for Mf in human. The lack of concordance between MX and Mf-surveys at the cluster level could either be due to migration of individuals from endemic districts or the difference in the location of the HHs selected for the surveys. Lack of concordance has been reported elsewhere where both the surveys were carried out in the same HHs [31]. Such household level comparisons may not be realistic as the mosquitoes trapped are not necessarily from the same house where the traps are placed and the fact that MX sampling is not designed to take a decision at a household or cluster level.

The challenge with respect to MX is the availability of adequate manpower (technical expertise in mosquito identification and molecular assays) and laboratory facilities for processing the samples in the health system. With current promotion of global vector control response by the WHO, capacity building and strengthening of laboratories can be done, which will not only be useful for MX but also cover all vector borne diseases [54]. Community-based Mf-survey is operationally more challenging and time-consuming (invasive blood sampling, inconvenient survey time, mandatory written consent from volunteered individuals) than MX survey. MX is non-invasive and requires only verbal consent for placing gravid traps in the selected HHs.

In school-based TAS, the schools are selected systematically with probability proportional to the size of the target population (no. of children in I and II grades) in schools. In this selection procedure, though it provides equal probability of selecting schools, schools with large strengths share a major fraction of the sampled children. In our study, of the 43 schools selected for TAS, 35 (81.4%) are government schools and 8 (18.6%) are private schools. The eight private schools with a large strength shared 56.2% of the children (n = 1578) selected for TAS compared to 43.8% from 35 government schools. The above percentages represented the actual distribution of schools (80% government schools and 20% private schools) and children (46.9 vs 53.0%) in the EU. Children in the private institutions are mostly from middle class and above. Most of the government schools are spatially located in rural areas spread over the entire EU preferred by students from a low socio-economic background and at higher risk of infection (in terms of exposure to LF infection) [55–57]. Therefore, school-based TAS is likely to miss antigen positives from the small and highly focal residual clusters of transmission if the prevalence of infection is spatially variable within an EU. On the other hand, as for Mf-survey, a community-based TAS is practically more challenging (house-to-house visit, time of survey depending on the availability of residents, numbering and locating houses and defining area boundaries) and time-consuming than a school-based TAS, though it could minimize the risk of failing to detect the antigen positives among the children from the areas at high risk of transmission. Further, both Mf-survey and TAS are invasive and require written consent for drawing blood sample from each volunteered individual in a household.

### Costs of MX and TAS

**Fig 6:**
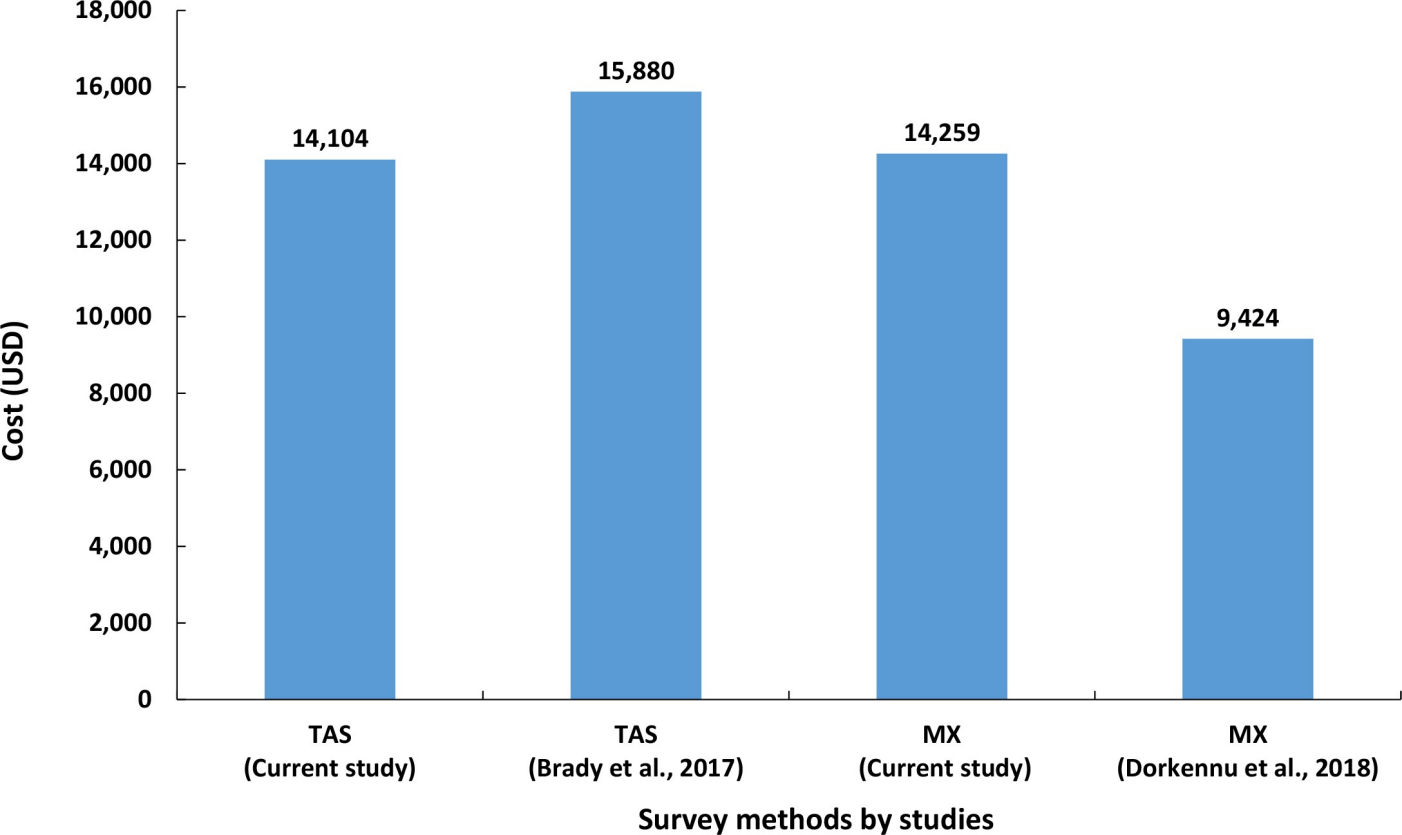
Comparison of total cost per evaluation unit for transmission assessment survey (TAS) or molecular xenomonitoring (MX) incurred by studies carried out in different countries.

In the present study, the cost of MX was estimated to be $14259 USD per EU for 358 pools (Fig 6). As has been reported elsewhere [20, 58], a major part of the MX-cost incurred was towards allowances and wages for the personnel followed by transportation costs. The cost of MX in this study was higher than the estimate reported in Togo, African region ($11970.13 USD per EU for 210 pools) [20] which is inclusive of the cost of $2546.5 USD towards consultancy, training, ethics application, vehicle maintenance, data management, communication and shipment of samples to the central laboratory. The cost per EU after excluding all the above components is $9423.6 USD (Fig 6). Thus, the cost of per pool collected and processed was $44.9 USD in the African study against $40.8 USD in the current study. Despite the use of low-cost LAMP assay ($0.82 USD per test) [59], the cost per pool in the African study was marginally higher than that in the present study using TE based quantitative PCR assay ($3 USD per test). The higher cost could be due to the different methods (pyrethrum spray catches, human landing and exit trap collections) employed for collecting *Anopheles* mosquito vectors compared to gravid traps used for *Culex* mosquito in the present study.

The cost of school-based TAS per EU ($14104 USD) in our study is lower than that derived ($16179 USD) for Asian countries (Fig 6) [58]. As ours is a research study, we did not include the cost of training the personnel. The cost in this study is less than the cost per EU for Asian region even after excluding the training component ($15880 USD). Alternatively, based on the findings of Brady et al. [58], had a community-based TAS been conducted in our study area, the estimated cost ($39971.0USD) would have been 2.83 times higher than that of the school-based TAS. If FTS were used instead of ICT, then the costs of school and community-based TAS per EU would be reduced by 43.6% and estimated to be $7941USD and $22,504USD, respectively. Though community-based TAS is more expensive than school-based TAS, it has been reported to be more sensitive in detecting residual hotspots [9]. However, the cost of community based TAS with FTS is 1.56 times higher than that of MX. Further, since MX is a non-invasive mosquito-based survey, and a more sensitive tool than school or community-based TAS in detecting residual hotspots in an EU, with a sample size powered enough to detect residual hotspots at a cluster-level, it could be a promising tool for remapping and monitoring transmission level during post MDA and validation phases. One of the limitations of the costing analysis is that this study was conducted in a research mode, which may not reflect the costing under programme, where there is scope for utilising the existing manpower after appropriate training, wherever available.

### Conclusions

The results of this study demonstrating the validity of MX at an evaluation unit level provide evidence to recommend MX as a tool complementary to TAS for stopping MDA and to detect resurgence of infection during post-MDA surveillance or validation phases. WHO is reviewing available evidence from research studies and current country experiences to develop specific post-validation surveillance guidelines [4]. Epidemiological indicators such as antibody (Ab) in the younger age classes, antigen (Ag) among adults and infection in vectors are considered by WHO for post-validation surveillance. Mf-prevalence survey requires large samples and visiting individuals at night. On the other hand, MX is feasible and can assess not only the ongoing risk of transmission but also residual transmission hotspots and the risk of resurgence of infection. MX can also provide information on vector abundance to plan vector management, if required. Early detection of transmission risk using MX will be useful to initiate appropriate measures of vector control to prevent resurgence of infection during post-MDA and validation phases.

## Supporting information

S1 Table Population, and reported MDA coverage in different years in Cuddalore district, Tamil Nadu, India. (DOCX)

S2 Table Independent assessment of MDA coverage in Cuddalore district, Tamil Nadu, India during 2014 by the ICMR-Vector Control Research Centre, Pondicherry. (DOCX)

S3 Table Results of microfilaria survey in sentinel, spot-check and additional random sites carried out in Cuddalore district, Tamil Nadu, India during 2014. (DOCX)
