# Supplementary material for: Molecular xenomonitoring as a post-MDA surveillance tool for global programme to eliminate lymphatic filariasis: Field validation in an evaluation unit in India
Source: PLoS Negl Trop Dis. 2020 Jan 24;14(1):e0007862. doi: 10.1371/journal.pntd.0007862 (PMC7001988; doi:10.1371/journal.pntd.0007862)
Supplement: S1 Table — (DOCX) [file pntd.0007862.s001.docx]

| **Table S1.** Population, and reported MDA coverage in different years in Cuddalore district, Tamil Nadu, India | | | | |
| --- | --- | --- | --- | --- |
| Year | Drugs | Population | No. treated | Reported coverage (%) |
| 1996 | DEC | 2065610 | 2003642 | 97.0 |
| 1997 | DEC | **Data not available** | | |
| 1998 | DEC | **Data not available** | | |
| 1999 | DEC | 2082266 | 1950504 | 93.7 |
| 2000 | DEC+albendazole | 2204869 | 2073107 | 94.0 |
| 2001 | DEC+albendazole | 2251729 | 2170667 | 96.4 |
| 2002 | DEC+albendazole | 2158192 | 2026430 | 93.8 |
| 2003 | DEC+albendazole | 2197655 | 2065893 | 94 |
| 2004 | DEC+Albendazole | 2237118 | 2105356 | 94.0 |
| 2005 | **MDA not conducted** | | | |
| 2006 | **MDA not conducted** | | | |
| 2007 | DEC+albendazole | 2093288 | 1998020 | 95.4 |
| 2008 | **MDA not conducted** | | | |
| 2009 | DEC+albendazole | 2141550 | 2057326 | 96.1 |
| 2010 | DEC+albendazole | 2126750 | 2095598 | 98.5 |
| 2011 | **MDA not conducted** | | | |
| 2012 | DEC+albendazole | 2247160 | 2169488 | 98.5 |
| 2013 | DEC+albendazole | **Data not available** | | |
| 2014 | DEC+albendazole | 2438217 | 2384903 | 97.8 |

MDA – Mass drug administration

DEC – Diethyl carbamazine

$$Reported coverage = \frac{No. treated}{Population} \times100$$
