# Supplementary material for: Molecular xenomonitoring as a post-MDA surveillance tool for global programme to eliminate lymphatic filariasis: Field validation in an evaluation unit in India
Source: PLoS Negl Trop Dis. 2020 Jan 24;14(1):e0007862. doi: 10.1371/journal.pntd.0007862 (PMC7001988; doi:10.1371/journal.pntd.0007862)
Supplement: S2 Table — (DOCX) [file pntd.0007862.s002.docx]

**Table S2.** Independent assessment of MDA coverage in Cuddalore district, Tamil Nadu, India during 2014 by the Vector Control Research Centre, Pondicherry

| Name of ward / Village selected for the survey | Number of persons interviewed | Number received drug | Drug coverage (%) | Number consumed drug | Consumption rate (%) |
| --- | --- | --- | --- | --- | --- |
| Thirumaaanikuzhi | 121 | 93 | 76.86 | 58 | 47.93 |
| Melpathi | 130 | 92 | 70.77 | 48 | 36.92 |
| Mudapuli | 137 | 114 | 83.21 | 87 | 63.50 |
| V.puthur | 128 | 97 | 75.78 | 84 | 65.63 |
| Cudddalore district | 516 | 396 | 76.74 | 277 | 53.68 |

MDA – Mass drug administration

$$Drug coverage = \frac{No. received drug}{No. of persons interviewed} \times100$$

$$Consumption rate = \frac{No. consumed drug}{No. of persons interviewed} \times100$$
