# Supplementary material for: Molecular xenomonitoring as a post-MDA surveillance tool for global programme to eliminate lymphatic filariasis: Field validation in an evaluation unit in India
Source: PLoS Negl Trop Dis. 2020 Jan 24;14(1):e0007862. doi: 10.1371/journal.pntd.0007862 (PMC7001988; doi:10.1371/journal.pntd.0007862)
Supplement: S3 Table — (DOCX) [file pntd.0007862.s003.docx]

**Table S3** Results of microfilaria survey in sentinel, spot-check and additional random sites carried out in Cuddalore district, Tamil Nadu, India during 2014

| **Year** | **Sites** | **BSE** | **No. Mf +ve** | **Mf prevalence %** |
| --- | --- | --- | --- | --- |
| 2014 | **Spot-check 1** | 500 | 0 | **0.00** |
| 2014 | **Spot-check 2** | 500 | 0 | **0.00** |
| 2014 | **Spot-check 2** | 500 | 0 | **0.00** |
| 2014 | **Spot-check 2** | **500** | **0** | **0.00** |
| 2014 | **Sentinel 1** | 515 | 5 | **0.97** |
| 2014 | **Sentinel 2** | **502** | **0** | **0.00** |
| 2014 | **Sentinel 3** | **506** | **0** | **0.00** |
| 2014 | **Sentinel 4** | **500** | **0** | **0.00** |
| 2014 | **Additional random site 1** | **500** | **0** | **0.00** |
| 2014 | **Additional random site 2** | **500** | **0** | **0.00** |
| 2014 | **Additional random site 3** | **500** | **0** | **0.00** |
| 2014 | **Additional random site 4** | **500** | **0** | **0.00** |
| 2014 | **Additional random site 5** | **500** | **0** | **0.00** |
| 2014 | **Additional random site 6** | **500** | **0** | **0.00** |
| 2014 | **Additional random site 7** | **500** | **0** | **0.00** |
| 2014 | **Additional random site 8** | **500** | **1** | **0.20** |
| 2014 | **Additional random site 9** | **500** | **0** | **0.00** |
| 2014 | **Additional random site 10** | **500** | **0** | **0.00** |
|  |  |  |  |  |
| BSE - Blood Smears Examined | |  |  |  |
| Mf- Microfilaria | |  |  |  |
